# Impact of normobaric hyperoxia on finger vasomotor and thermoperceptual responses to local cold in normothermic and mildly hypothermic individuals

**DOI:** 10.1007/s00421-025-05869-0

**Published:** 2025-06-29

**Authors:** Michail E. Keramidas, Heather M. Bowes, Maaike I. Moes, Ola Eiken, Mikael Gennser

**Affiliations:** 1 Division of Environmental Physiology, Department of Physiology and Pharmacology, Karolinska Institute, Berzelius väg 13, Solna, 171 65 Stockholm, Sweden; 2 Division of Environmental Physiology, Swedish Aerospace Physiology Center, KTH Royal Institute of Technology, Stockholm, Sweden; 3 Division of Research and Enterprise, University of New South Wales Sydney, Sydney, Australia; 4 Swedish Air Force, Air Component Command, Joint Medical Division, Uppsala, Sweden

**Keywords:** CIVD, Cold injury, Diving, Oxygen, Immersion, Skin blood flow, Thermal pain

## Abstract

**Aim:**

To evaluate whether a sustained period of normobaric O_2_ breathing would modulate acral-skin vasoreactivity and thermosensitivity to localised cooling.

**Methods:**

Eight healthy men performed a 30-min normoxic and hyperoxic (100% O_2_) hand cold (8 °C water) provocation, while immersed to the chest either in 35.1 (0.4)°C (normothermic trial) or in 21.0 (0.1)°C (hypothermic trial) water. Finger temperature, circulatory and perceptual responses were monitored.

**Results:**

During the normothermic trial, hyperoxia augmented the cold-induced drop in finger temperature, and attenuated the rewarming (*P* = 0.03). Hyperoxia also enhanced the sensation of pain (*P* = 0.04). During the hypothermic trial, hyperoxia did not modify finger temperature, circulatory and perceptual responses to cold (*P* > 0.05).

**Conclusion:**

In normothermia, hyperoxia aggravates finger cold-induced vasoconstriction and pain sensation. In mild hypothermia, however, any hyperoxia-evoked influence on finger vasomotion and thermonociception is overridden by the generalised vasoconstriction and thermal discomfort instigated by whole-body cooling.

## Introduction

Despite the use of advanced protective suits and gloves, divers in arctic and subarctic regions are at risk of developing freezing and non-freezing cold injury in the extremities (i.e., fingers, toes), while operating, for sustained periods, in cold water, or during the transition phase from/to water while being exposed to very low air temperatures (e.g., in a support vessel) (Leitch and Pearson 1978; Adolfson et al. 1984; Valaik et al. 1998; Laden et al. 2007; Zander and Morrison 2008; Jorum and Opstad 2019; Sullivan-Kwantes and Tikuisis 2023). For instance, Heil et al. (2016) have documented that, between 2002 and 2014, 149 British soldiers were diagnosed with a cold injury, and approximately 50% of them were Royal Marines, who mostly were injured during their Arctic training—similar results have also been obtained in the US Marine Corps (Maule et al. 2024).

During such underwater exposures, the capacity to preserve acral-skin temperature and blood-flow, is compromised, not only by the low ambient temperatures encountered, but also by the independent influence of breathing gases on cutaneous vasomotion (see Mekjavic et al. 2003; Mekjavic and Eiken 2006; Moes et al. 2023). Alterations in the partial pressure of metabolically active gases, such as oxygen (O_2_), may thus perturb the central and/or peripheral regulation of heat-conservation processes. For instance, inhalation of a gas mixture with reduced O_2_ tension (i.e., hypoxia) seems to enhance susceptibility to cold injury, since it accelerates body core cooling, thereby aggravating skin constrictor responsiveness to cold (Keramidas et al. 2019); and it impedes fingers’ reperfusion following local cold exposure, and blunts the regional sensation of coldness and discomfort compromising thermo-behavioural actions (Fahim 1992; Golja et al. 2004; Keramidas et al. 2014, 2019; Massey et al. 2018).

Aside from the risk of being subjected to a hypoxic condition during, for instance, a malfunction of the breathing equipment, underwater operators commonly breathe hyperoxic gas mixtures at great depths. Yet information on the impact of sustained exposure to high partial O_2_ pressure on the cutaneous responses to cold is relatively scarce (cf. Mekjavic et al. 2003). It is well documented that, in euthermic individuals, a brief period of normobaric 100% O_2_ administration reduces blood flow, probably through nonadrenergic mechanisms, in the acral and non-acral cutaneous beds (Bertuglia et al. 1991; Crawford et al. 1997; Mak et al. 2002; Rousseau et al. 2007; Yamazaki et al. 2007; Yamazaki 2007; Keramidas et al. 2012). Notwithstanding, previous works have not detected any hyperoxia-induced modifications in skin temperature during whole-body exposure to mild-to-moderate cold (Froese 1958; Maccanon and Eitzman 1961; Savic and Mekjavic 1994), whereas, to the best of our knowledge, no actual measurements of cutaneous blood flow have been conducted in such conditions. Interestingly, and conversely, the arterial-pressor response to local (hand) cold provocation appears to be amplified by short-term inhalation of O_2_-enriched air (Maccanon 1960; Joshi et al. 1973). Furthermore, a few (Goldschneider and Ehrmann 1924; Maccanon and Eitzman 1961), but not all (Froese 1958; Savic and Mekjavic 1994; Ferguson et al. 2018) studies have suggested that the sensation of coldness is blunted by hyperoxia.

Accordingly, this study aimed to assess whether a sustained period of pure O_2_ breathing in normobaria, would modulate acral-skin vasoreactivity and thermosensitivity to direct, localised cooling. Considering that the cutaneous responsiveness to cold is affected largely by the whole-body thermal state (Caldwell et al. 2014; Keramidas et al. 2019), finger temperature, circulatory and perceptual responses were monitored continually during a normoxic and a hyperoxic (i.e., 100% O_2_) hand cold (8 °C water) provocation trial, while subjects were either maintained normothermic or rendered mildly hypothermic. We hypothesised that high levels of arterial O_2_ tension would invariably augment finger vasoconstriction but mitigate the perception of coldness and discomfort produced by localised cold stimulation.

## Methods

### Ethics approval

The study was approved by the Human Ethics Committee of Stockholm (Ref. no.: 2021-00932) and conformed to the standards set by the *Declaration of Helsinki*. Subjects were informed in detail about the experimental procedure before giving their written consent to participate.

### Subjects

Eight healthy, right-handed men participated [mean (range): age 25 (21–30) years, body mass 81.8 (64.9–95.4) kg, height 180 (163–186) cm, total skinfold thickness 94 (50–143) mm, and right-hand volume 414 (326–459) mL]. The sample size was based on our previous work employing a similar experimental design (Keramidas et al. 2019), using *α* = 0.05, *β* = 0.85 and an effect size *f* = 0.50 (G*power software, Heinrich Heine-Universitat, Dusseldorf, Germany; Faul et al. 2007). Subjects were nonsmokers, normotensive, were not taking any medication, and had no history of cold injury. They were instructed to abstain from alcohol and strenuous exercise for at least 24 h before each trial, to refrain from caffeine during the testing day, and to maintain their sleeping, eating and exercise routines throughout the study period.

### Experimental protocol

The study used a repeated-measures, single-blinded design. Specifically, subjects performed, on four separate occasions (days), a local cold provocation consisting of a 30-min hand immersion in 8 °C water, while they were immersed to the chest in: (i) 35.1 (0.2)°C water and were inhaling normal air [fraction of inspired oxygen (F_I_O_2_): 0.21; i.e., *normothermic, normoxic* trial], (ii) 35.0 (0.5)°C water and were inhaling pure O_2_ (F_I_O_2_: 1.0; i.e., *normothermic, hyperoxic* trial), (iii) 21.0 (0)°C water and were inhaling normal air (i.e., *mild-hypothermic, normoxic* trial), and (iv) 21.0 (0.1)°C water and were inhaling pure O_2_ (i.e., *mild-hypothermic, hyperoxic* trial). To induce a normothermic state, the initial temperature of the water was set at 35.5 °C (Craig and Dvorak 1966; Keramidas et al. 2019); yet, during the course of each immersion, it was slightly adjusted so that the individual rectal temperature (*T*_*rec*_) remained relatively constant. For each thermal state, no differences were detected between the two breathing conditions with regards to the water temperature (normothermic trial: *P* = 0.43; hypothermic trial: *P* = 0.63). The four trials were conducted in a quasi-Latin-square fashion: they were performed in a counterbalanced order, but, for each breathing condition (i.e., normoxia and hyperoxia), the mild-hypothermic trial was performed first, because, in the normothermic trial the immersion period preceding the hand cold provocation was determined by the time required to induce a 0.5 °C drop in *T*_*rec*_ in the mild-hypothermic trial (Keramidas et al. 2019). The study was carried out between June and November. Yet, for the individual subject, the four trials, which were performed at the same time of the day, were completed within a 21-day period and were separated by ≥ 3 and ≤ 8 days. The mean (standard deviation; SD) temperature, relative humidity and barometric pressure in the laboratory were 27.5 (0.4)°C, 33 (6)% and 756 (7) mmHg, respectively.

Subjects were always clad in regular swim shorts. During all trials, they were equipped with an oronasal mask, and breathed through a low resistance two-way respiratory valve (Model 2, 700 T-Shape, Hans Rudolph, Inc. Shawnee, USA); the inspiratory side of the valve was connected via respiratory corrugated tubing to a bag filled with the respective pre-mixed humidified gas. Each trial commenced with a 20-min baseline phase, during which subjects remained in a resting, semi-reclining position on a gurney placed next to a tank. In the hyperoxic trials, the 20-min baseline phase comprised 10 min of breathing normoxic gas, followed by breathing 100% O_2_ until the end of the trial; presumably, the 10-min period in hyperoxia was sufficient to stabilise the arterial partial O_2_ pressure at an elevated level, as indicated by the slightly enhanced values of capillary oxyhaemoglobin saturation [SpO_2_; normoxia: 98 (1)%, hyperoxia: 100 (1)%; *P* < 0.001]. After the 20-min baseline phase, subjects entered the tank, which was filled with stirred water maintained at either 35.1 (0.4) °C (i.e., in the normothermic trials), or 21.0 (0.1)°C (i.e., in the mild-hypothermic trials). They were immersed to the level of the xiphoid process, and remained in a semi-upright sitting position with both arms being supported at the level of the heart, above the water surface. The left hand was exposed to the ambient room temperature throughout. The right hand (i.e., the test hand) was placed in a custom-made, water-perfused, tube-lined mitten, and warm water was circulated through the tubes maintaining the skin temperature of the fingers at 35.5–36.0 °C (KTH4H/B, Panasonic, Aichi, Japan) (Keramidas et al. 2022). The mitten was covering the entire hand, and the most distal portion of the forearm (i.e., it extended up to ~15 cm above the wrist). During the mild-hypothermic trials, subjects rested idle in this position until their *T*_*rec*_ dropped by 0.5 °C from baseline (B-WI phase). As mentioned previously, during the normothermic trials, the duration of B-WI phase was similar to that obtained in the mild-hypothermic trial of the respective breathing condition. At the end of each B-WI phase, the right hand was removed from the mitten, was covered with a thin plastic bag, and was immersed up to the ulnar and radial styloids for 30 min in a different tank filled with 8 °C water (H-CWI phase). After the completion of the H-CWI phase, the right hand was removed from the water, and a 15-min spontaneous hand rewarming period ensued (H-RW phase), during which subjects remained in the tank with both arms resting on the arm-support and being exposed to the room temperature. Afterwards, subjects were removed from the tank, placed in a well-insulated sleeping bag on the gurney, and were monitored for a further 30-min period (B-RW phase).

### Measurements

*T*_*rec*_ was monitored continuously with a rectal thermistor (Yellow Springs Instruments, Yellow Springs, OH) inserted ~10 cm beyond the anal sphincter. Mean skin temperature (*T*_*sk*_) was derived from the unweighted average of skin temperatures, recorded with copper-constantan (T-type) thermocouple probes (Physitemp Instruments Inc., Clifton, NJ) at the left side of the body: on the forehead, upper arm, upper and low back, forearm, ring finger, chest, abdomen, thigh, calf, foot, and big toe. Mean body temperature ($\overline{T }$_*b*_) was calculated using the equation: $\overline{T }$_*b*_ = 0.64 × *T*_*rec*_ + 0.36 × *T*_*sk*_ (Burton 1935; Colin et al. 1971; Lenhardt and Sessler 2006). Five additional thermocouples were attached to the middle of the palmar side of the distal phalanx of each finger of the right hand. The average (*T*_*F-avg*_), minimum, and maximum finger temperatures were calculated. A finger skin-temperature elevation of ≥1 °C lasting for ≥3 min was defined as a cold-induced vasodilatation (i.e., CIVD) response (Keramidas et al. 2019). The following CIVD-related parameters were also assessed: (i) the total number of CIVD events, (ii) the temperature amplitude, which was calculated as the difference between the lowest temperature recorded just before the CIVD and the highest temperature reached during the CIVD, and *(iii)* the duration of each CIVD event. All temperatures were sampled at 1 Hz with a NI USB-6215 data acquisition system, and processed with LabVIEW software (v. 2019, National Instruments, Austin, TX).

Systolic (SAP), diastolic (DAP), and mean (MAP) arterial pressures were measured continuously using finger photoplethysmography (Finometer, Finapres Medical Systems BV, Amsterdam, The Netherlands). The pressure cuff was placed around the middle phalanx of the left middle finger, and the reference pressure transducer positioned at the level of the heart. Heart rate (HR) was derived from the arterial pressure curves as the inverse of the interbeat interval. Local skin blood flux was assessed at a rate of 10 Hz on the palmar side of the distal phalanx of the right (i.e., immersed) index finger and on the dorsal side of the left (i.e., non-immersed) forearm by laser-Doppler flowmetry (VMS-LDF2; Moor Instruments, Axminster, UK) using optic probes (VP1/7; Moor Instruments, UK). Skin blood flux was reported as cutaneous vascular conductance (CVC), calculated as skin blood flux divided by MAP.

SpO_2_ was monitored with an earlobe pulse oximeter (Radical-7, Masimo, Irvine, CA, USA). During the baseline and just before the H-CWI phase, glucose concentration was measured from a finger capillary blood sample (Accu-Check, Aviva, Roche, Mannheim, Germany).

During the baseline, B-WI (at 10-min intervals), H-CWI (at minutes 1, 2, 3, 4, 5, and every 5 min thereafter), H-RW (at minutes 1, 5, 10, and 15), and B-RW (at 10-min intervals) phases, subjects were asked to provide ratings of their whole-body and right-hand thermal sensation (from 1*-cold* to 7*-hot*) and comfort (from 1*- comfortable* to 4*-very uncomfortable*). At the same time intervals, the general affective valence (from -5*-very bad* to + 5-*very good*), the perceived shivering intensity (from 1*-no shivering* to 4*-heavy shivering*), and the local (right hand) pain (from 0*-no pain* to 10*-unbearable pain*) were also assessed. Upon arrival at the laboratory during the first session, subjects were introduced to and thoroughly familiarised with all scales.

### Statistical analyses

Data are presented as the mean of each phase. Baseline values were calculated as the average of the final 10 min of the 20-min baseline phase. Normality of distribution for all datasets was assessed using the Shapiro–Wilk test. A two-way [thermal status (mild hypothermia vs normothermia) × breathing condition (normoxia vs hyperoxia)] repeated-measures analysis of variance (ANOVA) was used for all physiological variables. Mauchly’s test was conducted to assess the sphericity and, if necessary, the Greenhouse–Geiser *ɛ* correction was used to adjust the degrees of freedom. When an ANOVA revealed significant effects, multiple pairwise comparisons were performed with Newman-Keuls post hoc test. A paired sample Student’s *t*-test was used to assess differences in the duration of B-WI phase between the normoxic and hyperoxic trials. Differences in the incidence of CIVD events were determined with the Chi-square test. Differences in the perceptual responses were evaluated with Friedman’s test, followed by a Wilcoxon test. The relationship between local pain and finger skin temperature was assessed with a Spearman rank correlation coefficient (*r*_s_); where the magnitudes of correlations were interpreted qualitatively using Cohen’s scale: *r* < 0.1: trivial, 0.1–0.3: small, 0.3–0.5: moderate, and >0.5: large. Statistical analyses were conducted using Statistica 8.0 (StatSoft, Tulsa, OK), and figures were produced using Prism 10.0 (GraphPad Software Inc., San Diego, CA, USA). Unless otherwise stated, data are presented as mean values with (SD). The *α* level of significance was set a priori at 0.05.

## Results

Subjects completed all trials without any adverse effects. When subjects were asked, in the middle of each trial (i.e., 32 trials in total), to identify the breathing gas (the question posed was: *“what gas do you think you are breathing? normal air, hyperoxia, or you do not know?”*), they were indecisive in 15 trials, guessed incorrectly in 11 trials, and correctly in 6 trials (note: one subject was correct in all four trials; cf. Gennser et al. 2000).

Hyperoxia consistently increased SpO_2_ [mean (range) normoxia: 98 (97–99)%, hyperoxia: 100 (99–100)%; *P* < 0.001]. The duration of the B-WI phase did not differ between trials [mean (range) normoxia: 109 (30–160) min, hyperoxia: 121 (38–160) min; *P* = 0.28). Subjects remained euglycemic throughout [mean glucose values in the mild-hypothermic trials: 5.6 (0.5) mmol L^−1^, and in the normothermic trials: 5.9 (0.3) mmol L^−1^; *P* ≥ 0.45].

### Normothermic trials

**Fig. 1:**
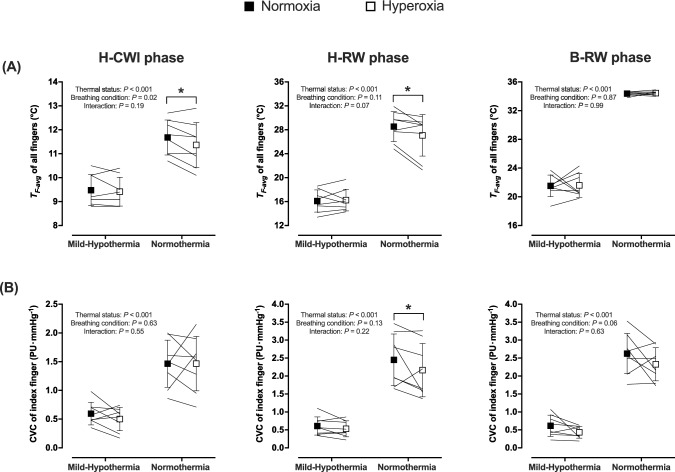
Mean (standard deviation) and individual skin temperature of all fingers (*T*_*F-avg*_; **A**) and cutaneous vascular conductance (CVC) of the index finger (**B**) of the right hand obtained during the 30-min hand cold-water immersion (H-CWI), the 15-min hand rewarming (H-RW), and the 30-min body rewarming (B-RW) phases in the mild-hypothermic and normothermic trials, while subjects were breathing either a normoxic (F_I_O_2_: 0.21) or a hyperoxic (F_I_O_2_: 1.0) gas mixture at atmospheric pressure. N = 8 healthy men. * Significant difference between normoxia and hyperoxia; *P* < 0.05

**Fig. 2:**
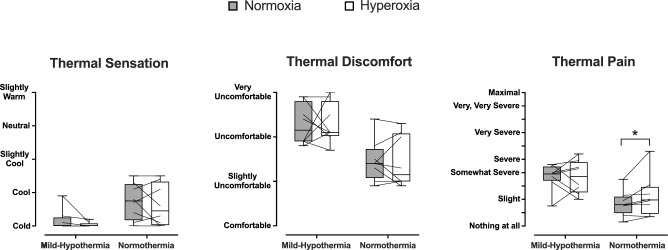
Box and whisker plots and individual data for the right-hand thermal sensation, comfort and pain obtained during the 30-min hand cold-water immersion phase in the mild-hypothermic and normothermic trials, while subjects were breathing either a normoxic (F_I_O_2_: 0.21) or a hyperoxic (F_I_O_2_: 1.0) gas mixture at atmospheric pressure. N = 8 healthy men. * Significant difference between normoxia and hyperoxia; *P* < 0.05

*T*_*rec*,_ *T*_*sk*_ and $\overline{T }$_*b*_ were similar in the two trials (*P* ≥ 0.55; Table 1). During the B-WI phase, *T*_*F-avg*_ was 36.0 (0.2)°C and 35.8 (0.2)°C in normoxia and hyperoxia, respectively (*P* = 0.21). During the H-CWI phase, hyperoxia aggravated the cold-induced drop in *T*_*F-avg*_ (*P* = 0.03; Fig. 1a), and reduced the minimum and maximum finger temperatures (*P* ≤ 0.02; Table 2). The finger cooling rate did not vary between the two H-CWIs [normoxia: −0.81 (0.02) °C min^−1^, hyperoxia: -0.81 (0.03)°C min^−1^; *P* = 0.44]. The number of CIVD events was similar in the two trials (*P* > 0.05; Table 2). During the H-RW phase, the finger-temperature rewarming was blunted by hyperoxia (*P* = 0.03; Fig. 1a). Hyperoxia did not alter finger CVC during the baseline phase [normoxia: 2.66 (0.58) PU mmHg^−1^, hyperoxia: 2.54 (0.60) PU mmHg^−1^; *P* = 0.65], but reduced it during the B-WI phase [normoxia: 3.14 (0.57) PU mmHg^−1^, hyperoxia: 2.55 (0.59) PU mmHg^−1^; *P* = 0.02]. No inter-trial differences were noted in finger CVC during the H-CWI (*P* = 0.96; Fig. 1b). Hyperoxia reduced finger CVC during the H-RW phase (*P* = 0.03), but not during the B-RW phase (*P* = 0.11) (Fig. 1b). Forearm CVC did not differ between trials throughout (*P* ≥ 0.07; Table 1). Hyperoxia enhanced SAP, DAP and MAP during the B-WI phase (*P* ≤ 0.04); no differences were noted in the other phases. HR was consistently lower in hyperoxia (*P* ≤ 0.03), although no post-hoc differences were detected (Table 1). Hyperoxia did not modify the whole-body perceptions (*P* > 0.05; Table 1), or the right-hand thermal sensation (*P* = 0.62) and comfort (*P* = 0.95) (Figure 2). Yet the pain sensation during the H-CWI phase was augmented by hyperoxia (*P* = 0.04; Figure 2). During the H-CWI phase, the right-hand pain was highly correlated with *T*_*F-avg*_, while subjects were either in hyperoxia (*r*_s_ = −0.73; *P* = 0.04), or in normoxia (*r*_s_ = −0.82; *P* = 0.01).

**Table 1:** Thermal, cardiovascular and perceptual responses obtained during the baseline, the body immersion (B-WI), the 30-min hand cold-water immersion (H-CWI), the 15-min hand rewarming (H-RW), and the 30-min body rewarming (B-RW) phases in the mild-hypothermic and normothermic trials, while subjects were breathing either a normoxic (F_I_O_2_: 0.21) or a hyperoxic (F_I_O_2_: 1.0) gas mixture at atmospheric pressure

	Normoxia					Hyperoxia				
	Baseline	B-WI	H-CWI	H-RW	B-RW	Baseline	B-WI	H-CWI	H-RW	B-RW
*Mild-Hypothermic trial*										
*T*_*rec*_ (°C)	37.2 (0.3)	37.1 (0.2)	36.7 (0.2)	36.5 (0.4)	36.4 (0.4)	37.3 (0.2)	37.2 (0.2)	36.8 (0.3)	36.7 (0.3)	36.5 (0.5)
Δ*T*_*rec*_ (°C)	0	−0.1 (0.1)	−0.5 (0.3)	−0.7 (0.5)	−0.8 (0.5)	0	−0.1 (0.1)	−0.5 (0.2)	−0.6 (0.3)	−0.8 (0.4)
*T*_*sk*_ (°C)	33.1 (0.5)	26.6 (0.4)	26.2 (0.5)	26.1 (0.5)	28.1 (0.7)	33.2 (0.6)	26.4 (0.7)	26.2 (0.4)	26.2 (0.4)	28.0 (0.6)
$\overline{ T }$_*b*_ (°C)	35.7 (0.1)	33.4 (0.2)	32.9 (0.2)	32.8 (0.2)	33.4 (0.3)	35.8 (0.2)	33.3 (0.3)	33.0 (0.2)	32.9 (0.3)	33.5 (0.4)
Forearm CVC (PU mmHg^−1^)	0.35 (0.29)	0.27 (0.18)	0.21 (0.10)	0.24 (0.13)	0.16 (0.07)	0.35 (0.17)	0.25 (0.11)	0.27 (0.24)	0.23 (0.17)	0.19 (0.12)
SAP (mmHg)	136 (14)	136 (13)	145 (13)	140 (11)	143 (12)	126 (7)	137 (9)	146 (9)	140 (10)	141 (13)
DAP (mmHg)	84 (9)	82 (8)	89 (9)	87 (7)	89 (8)	78 (5)	83 (6)	92 (7)	89 (10)	91 (7)
MAP (mmHg)	101 (10)	100 (10)	107 (10)	105 (8)	107 (9)	94 (5)	101 (7)	110 (7)	106 (9)	108 (8)
HR (beats min^−1^)	79 (13)	67 (13)	65 (14)	65 (14)	60 (12)	74 (18)	65 (15)	63 (14)	60 (12)*	58 (13)
Body thermal sensation	4.7 (4–6)	2.3 (2–4)	2.4 (1–4)	2.4 (1–4)	3.6 (2–5)	4.4 (4–6)	2.3 (2–4)	2.6 (1–4)	2.3 (1–4)	3.5 (3–4)
Body thermal comfort	1	1.9 (1–3)	2.1 (1–4)	2.2 (1–4)	1.3 (1–2)	1	1.9 (1–2)	2.0 (1–3)	2.1 (1–3)	1.2 (1–2)
Affective valence	2.4 (0–5)	1.9 (0–4)	−0.1 (−1 to 3)	1.1 (−1 to 4)	1.6 (0–4)	1.8 (0–4)	1.5 (0–4)	−0.6 (−2 to 4)	0.7 (−1 to 4)	1.7 (0–4)
Perceived shivering	1	1.4 (1–2)	2.2 (1–4)	2.1 (1–4)	1.3 (1–3)	1	1.5 (1–3)	2.2 (1–4)	2.1 (1–4)	1.2 (1–2)
*Normothermic trial.*										
*T*_*rec*_ (°C)	37.3 (0.3)	37.2 (0.2)	37.3 (0.2)	37.3 (0.2)	37.3 (0.2)	37.3 (0.2)	37.3 (0.1)	37.3 (0.2)	37.3 (0.3)	37.3 (0.3)
Δ*T*_*rec*_ (°C)	0	0 (0.1)	0 (0.1)	0 (0.1)	0 (0.1)	0	0 (0.2)	0 (0.2)	0 (0.2)	0 (0.2)
*T*_*sk*_ (°C)	33.3 (0.7)	34.7 (0.3)	34.3 (0.4)	34.2 (0.4)	34.1 (0.3)	33.2 (0.6)	34.4 (0.3)	34.2 (0.6)	34.1 (0.5)	34.1 (0.4)
$\overline{ T }$_*b*_ (°C)	35.8 (0.3)	36.3 (0.2)	36.2 (0.2)	36.2 (0.3)	36.1 (0.2)	35.8 (0.1)	36.3 (0.1)	36.2 (0.3)	36.1 (0.2)	36.1 (0.3)
Forearm CVC (PU mmHg^−1^)	0.60 (0.79)	1.15 (1.04)	0.95 (0.82)	0.96 (0.85)	0.60 (0.84)	0.55 (0.23)	0.63 (0.24)	0.58 (0.22)	0.61 (0.26)	0.38 (0.15)
SAP (mmHg)	131 (13)	114 (10)	126 (12)	127 (16)	130 (12)	135 (7)	127 (8)*	135 (11)	134 (12)	140 (8)
DAP (mmHg)	81 (7)	70 (7)	76 (10)	76 (11)	79 (11)	85 (5)	76 (5)*	79 (7)	78 (8)	85 (6)
MAP (mmHg)	98 (8)	85 (7)	93 (10)	93 (12)	96 (11)	101 (5)	93 (6)*	98 (8)	96 (9)	103 (6)
HR (beats min^−1^)	76 (9)	75 (8)	71 (10)	70 (11)	72 (11)	73 (8)	71 (8)	68 (10)	69 (10)	69 (9)
Body thermal sensation	4.4 (4–5)	4.9 (4–6)	4.3 (4–5)	4.3 (4–5)	4.4 (4–5)	4.8 (4–6)	5.1 (4–6)	4.4 (4–6)	4.4 (4–6)	4.6 (4–6)
Body thermal comfort	1	1	1	1	1	1	1	1	1	1
Affective valence	1.9 (0–4)	2.3 (0–4)	1.6 (−1–4)	2.1 (0–4)	2.1 (0–4)	2 (0–4)	2.1 (0–4)	1.2 (0–4)	1.9 (0–4)	2.2 (0–4)
Perceived shivering	1	1	1	1	1	1	1	1	1	1

Values are mean (standard deviation) for rectal (*T*_*rec*_), skin (*T*_*sk*_) and mean body ($\overline{T }$_*b*_) temperatures, forearm cutaneous vascular conductance (CVC), systolic (SAP), diastolic (DAP) and mean (MAP) arterial pressures, and heart rate (HR). Data are presented as the mean for the entire phase, aside from the baseline values, which are the mean of the final 10 min of the 20-min baseline phase. Values are mean (range) for thermal sensation (from 1*-cold* to 7*-hot*), thermal comfort (from 1*- comfortable* to 4*-very uncomfortable*), affective valence (from −5*-very bad* to +5-*very good*), and perceived shivering (from 1*-no shivering* to 4*-heavy shivering*). N = 8 healthy men. * Significant difference between normoxia and hyperoxia; *P* < 0.05

**Table 2:** Minimum and maximum finger temperature, number of cold-induced vasodilation (CIVD) events, temperature amplitude and duration of cold-induced vasodilatation on the palmar side of the distal phalanx of the right-hand fingers during the 30-min hand cold-water immersion phase in the mild-hypothermic and the normothermic trials, while subjects were breathing either a normoxic (F_I_O_2_: 0.21) or a hyperoxic (F_I_O_2_: 1.0) gas mixture at atmospheric pressure

	Mild-Hypothermic trial		Normothermic trial	
	Normoxia	Hyperoxia	Normoxia	Hyperoxia
Minimum temperature (°C)	8.3 (0.5)	8.2 (0.4)	9.2 (0.7)	8.9 (0.5)*
Maximum temperature (°C)	14.6 (1.5)	13.6 (1.7)	18.1 (2.2)	16.4 (2.2)*
CIVD events (no.)	1/5	1/5	7/56	6/52
CIVD amplitude (°C)	1.6 (0.2)	1.0 (0.1)	3.0 (1.1)	3.2 (1.4)
CIVD duration (min)	8.6 (2.1)	10.1 (0.2)	7.2 (1.7)	8.8 (2.5)

Values for minimum and maximum temperatures, and amplitude and duration of CIVD are mean (standard deviation). Values for CIVD events are mean/total incidence. N = 8 healthy men

*Significant difference between normoxia and hyperoxia

### Mild-hypothermic trials

*T*_*rec*_, *T*_*sk*_ and $\overline{T }$_*b*_ dropped (*P* < 0.001) to a similar extent during the course of the two trials (*P* ≥ 0.40; Table 1). During the B-WI phase, *T*_*F-avg*_ was 35.7 (0.2)°C and 35.6 (0.2)°C in normoxia and hyperoxia, respectively (*P* = 0.71). During the H-CWI and rewarming phases, *T*_*F-avg*_ did not differ between trials (*P* ≥ 0.58; Fig. 1a). The rate of finger cooling was similar in the two H-CWIs [normoxia: −0.87 (0.02)°C min^−1^, hyperoxia: −0.87 (0.02)°C min^−1^; *P* = 0.92]. No inter-trial differences were noted in the minimum and maximum finger temperature, nor in the incidence, amplitude, and duration of the CIVD events (*P* > 0.05; Table 2). Hyperoxia did not modify finger CVC during the baseline [normoxia: 2.68 (0.95) PU mmHg^−1^, hyperoxia: 2.43 (0.36) PU mmHg^−1^; *P* = 0.77] and B-WI [normoxia: 1.24 (0.78) PU mmHg^−1^, hyperoxia: 0.89 (0.31) PU mmHg^−1^; *P* = 0.11] phases, or during the H-CWI and rewarming phases (*P* ≥ 0.30; Fig. 1b). Forearm CVC was similar across the two trials throughout (*P* ≥ 0.82; Table 1). Hyperoxia did not alter SAP, DAP and MAP (*P* > 0.05; Table 1), but consistently attenuated HR, especially during the H-RW phase (*P* = 0.05; Table 1). Neither the whole-body (Table 1) nor the regional (i.e., right-hand; Figure 2) perceptual responses were modified by hyperoxia (*P* > 0.05). During the H-CWI phase, the right-hand pain sensation was highly correlated with *T*_*F-avg*_, while subjects were in hyperoxia (*r*_s_ = −0.84; *P* = 0.01), but not in normoxia (*r*_s_ = −0.60; *P* = 0.12).

## Discussion

Using a within-subject design, the present study sought to examine, in healthy noncold-acclimatised men, the impact of sustained exposure to normobaric hyperoxia on acral-skin responsiveness to localised cooling. To this end, finger temperature, circulatory and perceptual responses were monitored during and after a 30-min hand immersion in 8 °C water, while subjects were inhaling, in a single-blinded manner, either a normoxic (F_I_O_2_: 0.21), or a hyperoxic (F_I_O_2_: 1.0) gas mixture, during the course of two distinctly different whole-body thermal states, that is, in normothermia (*T*_*rec*_ ~ 37.3 °C, *T*_*sk*_ ~ 34 °C) and mild hypothermia (*T*_*rec*_ ~ 36.7 °C, *T*_*sk*_ ~ 26 °C). The main outcomes of the study were that: (i) in normothermic conditions, the inhalation of pure O_2_ aggravated the magnitude of finger vasoconstriction and pain sensation instigated by local cold, and transiently delayed digits’ spontaneous rewarming after the removal of the cold stress, and (ii) in hypothermic conditions, any hyperoxia-specific influence on finger vasomotion and thermonociception was masked by the generalised vasoconstriction and thermal discomfort produced by whole-body cooling.

Prior to the immersion of the hand in 8 °C water, finger skin temperature was deliberately clamped at ~36 °C in all four trials. Hyperoxia, nevertheless, reduced finger CVC by ~19% during the normothermic trial. Similarly, the forearm CVC elevation triggered by the 35.5 °C-water immersion (Arborelius et al. 1972; Gabrielsen et al. 2000; Keramidas et al. 2019) was blunted by pure O_2_: it increased by ~143% and ~36% in the normoxic and the hyperoxic B-WI phases, respectively (*P* = 0.01). These findings did, in fact, confirm that, in euthermic individuals, hyperoxia constricts cutaneous blood vessels in both glabrous and non-glabrous areas (Bertuglia et al. 1991; Crawford et al. 1997; Mak et al. 2002; Rousseau et al. 2007; Yamazaki 2007; Yamazaki et al. 2007; Keramidas et al. 2012).

A novel finding of this study, however, was that the constrictor influence of O_2_ prevailed during the application of intense localised cold stimulus, as indicated by the lower *T*_*F-avg*_ values reached in the normothermic H-CWI phase (index-finger CVC was, on average, reduced by ~15% and increased by ~56% in six and two subjects, respectively); whereas the incidence of CIVD response remained unaltered. Hyperoxia also delayed (only in H-RW) the temperature and blood-flow recovery of the fingers following the local cold stimulation. We are unable to, by the current experimental design, identify the mechanisms underlying this response. Yamazaki et al. (2007), who employed regional pharmacological treatments, have shown that, at least in the non-glabrous skin areas, the hyperoxic vasoconstriction was mediated primarily through nonadrenergic mechanisms; the basal activity of nitric oxide synthase was thus inhibited during pure O_2_ inhalation. Arguably, the drop in finger CVC observed in our study, could also be ascribed to a hyperoxia-induced alteration in the reflex neural control of cutaneous vasomotion, given that, in this breathing condition, MAP was consistently higher by ~5–8 mmHg. Such an explanation seems less likely, however, because the magnitude of the systemic pressor increase in response to local cold provocation was similar in the normoxic (ΔMAP: 8 mmHg) and hyperoxic (ΔMAP: 5 mmHg) trials (*P* = 0.28). Yet, we cannot exclude that the hyperventilation, commonly evoked by hyperoxic breathing (due to the Haldane effect; Shock and Soley 1940; Dripps and Comroe Jr 1947; Becker et al. 1996), may have contributed partly to the finger vasoconstriction (Brown 1953; Delius et al. 1972). Regardless of the exact mechanism, present findings demonstrate that, in euthermic individuals, hyperoxia acts to constrict the acral skin vessels, with and without superimposition of direct local cold stress.

Contrary to in the normothermic trials, pure O_2_ breathing did not modulate cutaneous vasomotor reactivity, when subjects were mildly hypothermic. In this thermal condition, and regardless of the gaseous environment, the intensity of vasoconstriction was aggravated and the incidence and magnitude of CIVD events were consistently reduced in the finger arterioles during the local cold provocation. Evidently, the upregulation of peripheral sympathetic tone evoked by the whole-body cooling, overrode any independent effects of high O_2_ tension on skin vasculature. That the peripheral vasoconstriction induced by mild hypothermia tends to override the arterial partial O_2_ pressure effects on cutaneous vascular tone is in line with previous findings during sustained exposure to a low O_2_ tension environment (Keramidas et al. 2019). Collectively, our results substantiate and extend previous evidence (Spealman 1945; Daanen and Ducharme 1999; Brajkovic et al. 2001; Flouris and Cheung 2009; Caldwell et al. 2014; Keramidas et al. 2019) that, in a hypothermic state, the control of cutaneous vasomotor activity is dictated wholly by the powerful and centrally driven constrictor tone, in the face of any concomitant manipulation in the partial pressure of inspired O_2_.

Aside from the autonomic functions, subjects’ whole-body thermal state also governed the thermoperceptual responses to local cold. The 8 °C-water was thus perceived colder, and more thermally uncomfortable and painful, while subjects were rendered mildly hypothermic. Hyperoxia, irrespective of the thermal condition, did not exert any prominent influence on the regional and whole-body discriminative and hedonic sensations. Yet, in normothermia, the inhalation of 100% O_2_ amplified the sensation of pain engendered by hand cold stimulation. The augmented nociceptive response was most likely driven by the lower skin-temperature of the fingers obtained in this condition; such an association was, indeed, corroborated by the high negative correlation coefficient estimated between the *T*_*F-avg*_ values and the self-reported pain ratings. The correspondence between the perceived pain severity and the actual thermal status of the hand, suggests that hyperoxia did not interfere with the function of hand-skin receptors, or the transmission and integration of thermoafferent cues. Therefore, and considering that even higher-order cognitive abilities (e.g., attention, memory) seem to remain intact whilst breathing 100% O_2_ (Andersson et al. 2002), it is plausible that, in normobaric hyperoxic circumstances, the ability to thermoregulate behaviourally is preserved; this notion, however, needs to be evaluated directly.

During the 21 °C-water immersion, hyperoxia did not modify the rate of body-core cooling; a finding that concurs with that from other investigations (Froese 1958; Maccanon and Eitzman 1961; Savic and Mekjavic 1994). It is, however, noteworthy that two previous studies have shown that, during short-term exposure to 9–10 °C air, the cold-induced elevation in metabolic heat production was somewhat blunted by sustained (Froese 1958) and intermittent (Maccanon and Eitzman 1961) inhalation of pure O_2_. Still, in both these studies, the metabolic downregulation did not lead to a more profound hypothermic response; apparently, because the deficit in heat production was counterbalanced by enhanced heat conservation, as indicated by the lower skin temperatures in hyperoxia (Maccanon and Eitzman 1961). In the present study, unfortunately, no direct measurements of thermogenesis were conducted. Based on subjects’ self-reports, similar degrees of shivering tremor were perceived in both breathing conditions. Given that neither surface temperatures and forearm CVC (i.e., indexes of heat conservation output), nor body core temperature varied between the two hypothermic trials, we speculate that the heat-producing thermoeffector activity also remained unaffected by hyperoxia. In support of this, Savic and Mekjavic (1994) did not observe any O_2_-related influence on shivering and skin temperatures during the course of a 60-min 20 °C-water immersion.

In the present study, we used immersion in moderately cold water to provoke, in a controlled manner and relatively briefly, a mild hypothermic state (Keramidas et al. 2019). To account for any confounding influence of the immersion per se on peripheral vasomotion (Arborelius et al. 1972; Gabrielsen et al. 2000; Keramidas et al. 2019), the normothermic local-provocation trials were conducted during submersion in thermoneutral (i.e., 35.0–35.5 °C) water (Craig and Dvorak 1966). However, if, or to what extent, finger vasoreactivity to hyperoxia would be similar during longer and/or more severe cold-water immersion, as well as during cold-air exposure needs to be examined. Future work should also evaluate whether differences in cutaneous responses to hyperoxia exist with regards to sex, age, health and fitness-level status, and the level of cold acclimatisation. We, lastly, acknowledge that the present findings are delimited to the upper-limb skin vessels, and it remains unclear if they can be extrapolated to other cutaneous vascular beds.

## Conclusions

Present results demonstrate that, in normothermic individuals, sustained inhalation of 100% O_2_ at atmospheric pressure, aggravates finger constrictor and pain responsiveness to hand cold provocation. These hyperoxia-evoked vasomotor and nociceptive responses to local cooling, however, appear to be overridden entirely by the generalised vasoconstriction and thermal discomfort instigated by mild hypothermia. This information might be helpful to customise effective safety measures for diving operations in arctic and subarctic regions. Future studies are required to determine the mechanisms underlying the impact of high partial O_2_ pressure on cutaneous vasomotion during cold exposure.

## Data Availability

The data that support the findings of this study are available on reasonable request from the corresponding author. The data are not publicly available due to privacy or ethical restrictions.

## Abbreviations

B-RW: Body rewarming
B-WI: Body water immersion
CVC: Cutaneous vascular conductance
CIVD: Cold-induced vasodilatation
DAP: Diastolic arterial pressure
F_I_O_2_: Fraction of inspired oxygen
H-CWI: Hand cold water immersion
HR: Heart rate
H-RW: Hand rewarming
MAP: Mean arterial pressure
O_2_: Oxygen
SAP: Systolic arterial pressure
SpO_2_: Capillary oxyhaemoglobin saturation
$\overline{T }$_*b*_: Mean body temperature
*T*_*F-avg*_: Average finger skin temperature
*T*_*rec*_: Rectal temperature
*T*_*sk*_: Skin temperature
